# Prevalence of *Plasmodium falciparum* anti-malarial resistance-associated polymorphisms in *pfcrt*, *pfmdr1* and *pfnhe1* in Muheza, Tanzania, prior to introduction of artemisinin combination therapy

**DOI:** 10.1186/s12936-015-0642-2

**Published:** 2015-03-25

**Authors:** Nahla B Gadalla, Gloria Tavera, Jianbing Mu, Edward R Kabyemela, Michael Fried, Patrick E Duffy, Juliana M Sá, Thomas E Wellems

**Affiliations:** Laboratory of Malaria and Vector Research, National Institute of Allergy and Infectious Diseases, NIH, Rockville, MD USA; Muhimbili University of Health and Allied Sciences, Dar es Salaam, Tanzania; Laboratory of Malaria Immunology and Vaccinology, National Institute of Allergy and Infectious Diseases, NIH, Rockville, MD USA; Case Western Reserve University School of Medicine, Cleveland, OH 44106 USA

**Keywords:** Malaria, Drug resistance, Chloroquine, Amodiaquine, Monodesethylamodiaquine, Quinine

## Abstract

**Background:**

A report of the chloroquine and amodiaquine resistance *pfcrt*-SVMNT haplotype in Tanzania raises concern about high-level resistance to the artesunate-amodiaquine combination treatment widely employed in Africa. Mutations in the *pfmdr1* multi-drug resistance gene may also be associated with resistance, and a highly polymorphic microsatellite (ms-4760) of the *pfnhe1* gene involved in quinine susceptibility has not been surveyed in Tanzania.

**Methods:**

A total of 234 samples collected between 2003 – 2006 from an observational birth cohort of young children in Muheza, Tanzania were analysed. In these children, 141 cases of *P. falciparum* infections were treated with AQ and 93 episodes were treated with QN. Haplotypes of *pfcrt* and *pfmdr1* were determined by a Taqman assay, and ms-4760 repeats in *pfnhe1* were assessed by nested PCR amplification and direct sequencing. Parasite population diversity was evaluated using microsatellite markers on five different chromosomes.

**Results:**

The *pfcrt-*CVIET haplotype was present alone in 93.6% (219/234) of the samples over the study period; the wild-type chloroquine- and amodiaquine-sensitive haplotype *pfcrt*-CVMNK was present in 4.3% (10/234) of the samples; and both haplotypes were present in 2.1% (5/234) of the samples. No significant change in wild-type *pfcrt*-CVMNK prevalence was evident over the 4-year period of the study. The *pfcrt*-SVMNT haplotype associated with high-level amodiaquine resistance was not detected in this study. The *pfmdr1* locus was genotyped in 178 of these samples. The *pfmdr1-*YYNY haplotype predominated in 67.4% (120/178) of infections and was significantly associated with the *pfcrt-*CVIET haplotype. All samples carried the wild-type *pfmdr1*-N1042 codon. The ms-4760 repeat on *pfnhe1* locus displayed 12 distinct haplotypes with ms-4760-1 predominating in the population. Analysis of these haplotypes showed no association of a particular haplotype with quinine treatment outcome.

**Conclusion:**

The *pfcrt*-CVIET chloroquine resistance haplotype dominated in the collection of *P. falciparum* samples from Muheza. The *pfcrt*-SVMNT haplotype, which threatens the efficacy of amodiaquine and was reported in the same time period from Korogwe, Tanzania, 40 Km from Muheza, was not detected. Relative low prevalence of *pfcrt*-SVMNT in Africa may result from genetic or other factors rendering *P. falciparum* less supportive of this haplotype than in South America or other regions.

**Trial registration:**

Trial Protocol Number: 08-I-N064.

**Electronic supplementary material:**

The online version of this article (doi:10.1186/s12936-015-0642-2) contains supplementary material, which is available to authorized users.

## Background

Parasite resistance to anti-malarials including artemisinins [1,2] poses a great challenge to *Plasmodium falciparum* malaria control efforts. Monitoring parasite haplotypes that predict susceptibility to major anti-malarials can guide treatment policies. Chloroquine (CQ) and amodiaquine (AQ) resistance are linked to mutations in the *P. falciparum* CQ resistance transporter gene *pfcrt* [3-5]. Two major haplotypes associated with these forms of drug resistance are *pfcrt*-CVIET in Africa and regions of Asia, and *pfcrt*-SVMNT in regions of South America, Asia and The Pacific. The *pfcrt*-CVIET haplotype swept the African continent in the wake of widespread CQ use [6].

Evidence suggests that the prevalence of the *pfcrt*-CVIET haplotype has subsided with repopulation of CQ-sensitive *pfcrt*-CVMNK parasites in several regions where sulphadoxine-pyrimethamine (SP) or artemisinin-based combination therapy (ACT) replaced CQ as drugs recommended for the treatment of *P. falciparum* malaria [7-20]. This phenomenon has not been observed in South America where, despite removal of CQ and AQ, mutant *pfcrt*-SVMNT parasites remain highly prevalent [21]. A possible explanation for the faster declines of *pfcrt*-CVIET prevalence in Africa and Southeast Asia is that *pfcrt*-CVIET is less fit than *pfcrt*-SVMNT relative to wild-type *pfcrt*-CVMNK in the absence of drug pressure [22]. However, much remains to be understood about parasite fitness and drug resistance [23].

Polymorphisms in another transporter gene (*pfmdr1*) encoding P-glycoprotein homologue 1 (PfPgH-1, also known as PfMDR1) have also been associated with *P. falciparum* responses to AQ and its major metabolite, monodesethylamodiaquine (MDAQ), as well as to other drugs including artemisinin derivatives [24-26]. *In vitro* drug responses from Nigerian isolates suggest that the *pfmdr1*-86Y codon polymorphism may be associated with decreased susceptibility to AQ [27]. This same polymorphism together with *pfmdr1*-1246Y has been associated with AQ treatment failures [28-30]. Other studies found an increased frequency of the *pfmdr1-*YYY triplet (codon positions 86, 184, 1246) in recrudescent and recurrent infections after treatment with AQ [30-33] or artesunate-amodiaquine (AS/AQ) [34].

Quinine (QN) remains an efficacious alternative treatment for malaria, including severe malaria and malaria in pregnancy. QN resistance is not widespread in malaria endemic areas possibly due to the multigenic nature of its resistance mechanism. Polymorphisms in both *pfcrt* [4,35] and *pfmdr1* [25,36] have been associated with QN susceptibility *in vitro.* The *P. falciparum* homologue sodium-hydrogen ion exchanger gene (*pfnhe1*) located in chromosome 13 has been linked to moderate QN resistance: polymorphisms in one of three microsatellite regions of *pfnhe1*, namely two or more repeats of DNNND in ms-4760, were associated with a higher QN IC_50_ in the progeny of a genetic cross (HB3 × Dd2), in culture-adapted clinical isolates and in laboratory lines [37]. Data from patient isolates tested *in vitro* for their QN response showed an association of two or more DNNND of the *pfnhe1*-ms-4760 with higher IC_50_ values in some [38-41] but not all studies [42-44]. In a review of the genetic markers of QN resistance, Okombo and colleagues have grouped the ms-4760 into blocks of particular repeats (block I to VI) [45]. A recent worldwide analysis of ms-4760 sequences identified a high diversity of haplotypes as well as an apparent absence of some of these haplotypes from endemic regions of Africa [46].

Reports of the *pfcrt*-S_agt_VMNT (AGT codon for serine) haplotype in Tanzania [47] and Cameroon [48], in addition to the *pfcrt*-S_tct_VMNT (TCT codon for serine) haplotype in Angola [49], raise concern whether AQ treatment failures may increase, particularly if the *P. falciparum* parasites also carry *pfmdr1* N86 and 1042D codons that, together with *pfcrt*-SVMNT, are associated with the high levels of AQ resistance found in South America [5]. Spread of the *pfcrt*-SVMNT and *pfmdr1* N86/1042D combination would pose a major threat to AS/AQ combination currently used in 25 African countries for uncomplicated malaria [50]. In light of these reports, a survey for the presence of *pfcrt*-SVMNT and/or *pfmdr1* N86/1042D in samples collected from children treated for malaria in Muheza, located approximately 40 kilometres west of Korogwe District where the observation of *pfcrt*-SVMNT was reported [47], was undertaken in this study. In addition, the diversity of *pfnhe1* ms-4760 in children treated with QN in this holoendemic region of Tanzania was investigated.

## Methods

### Clinical samples

**Figure 1:**
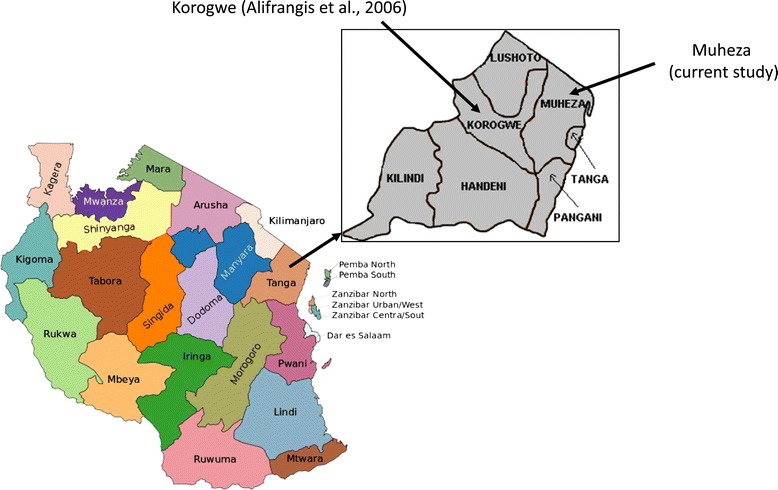
**Geographic locations of Muheza and Korogwe.** Map of Tanzania showing Tanga Region. Inset; the locations of Muheza District (present report) and Korogwe District where the presence of *pfcrt*-SVMNT was previously reported [47].

Samples analysed in this study had been collected from children aged 3 – 33 months as part of the Mother-Offspring Malaria Study performed in Muheza, northeastern Tanzania, between 2003 and 2006 (Figure 1). The details of this observational birth cohort study were approved by Tanzanian and US ethical review boards, and signed informed consent was obtained from all mothers for children to participate in the study, as previously reported [51,52]. Confirmation of infection with *P. falciparum* was performed by microscopic examination of blood smears. Blood samples from children who presented with symptoms consistent with clinical malaria (headache, nausea, vomiting, temperature ≥ 37.5°C) were stored on filter paper (FP). Parasite levels in the blood were reported as the number of parasites (pa) per 200 white blood cells (pa/200 WBC), and parasitaemia (pa/μl) estimates were calculated by assuming an average of 8000 WBCs/μl. Standard treatment for children with less than 2,500 pa/200 WBC was a 3-day 25-mg/kg AQ regimen (10, 10, 5 mg/kg/day). Children who failed AQ treatment or whose parasitaemia was above 2,500 pa/200 WBC were treated with QN at 10 mg/kg every 8 hours for 7 days [51]. Children unable to take the drugs orally were treated with intravenous QN twice daily until the child was able to take QN orally and complete the 7-day regimen.

### DNA extraction and genotyping

An area of approximately 7 mm^2^ from FP containing dry blood was cut with a pair of scissors that were decontaminated with 70% ethanol between samples. The FP was soaked overnight in 20% Chelex®. The supernatant was removed to a clean tube and the FP was washed twice in 1 ml 1X PBS. DNA was extracted using the QIAamp DNA mini kit (Qiagen Inc., USA) per the manufacturer’s recommendations and eluted in 50 μl of AE elution buffer. Samples from a non-template control (NTC) obtained by spotting parasite negative AB+ human blood on FP and from a piece of FP not containing any blood were included in each extraction batch (30 samples) to assure absence of contamination.

To assess the *pfcrt* haplotypes (CVMNK, CVIET, or S_agt_VMNT codons at positions 72–76), a Taqman assay was performed in a 25 μl volume containing 12.5 μl of Rotor-Gene Probe PCR Kit (Qiagen Inc., USA), 0.3 μM of each primer (PfCRT-D1 and PfCRTD2), 0.1 μM of each probe (crt76-CVMNK, crt76-CVIET, crt76-S_agt_VMNT) and 5 μl of sample DNA as previously described [53]. In addition, a probe that detects the S_tct_VMNT haplotype (crt76-S_tct_VMNT 5′- TCT GTA ATG AAT ACA ATT TTT GCT AA-3′) was designed for this study.

*Pfmdr1* haplotypes (codons 86, 184, 1042, and 1246) were determined by a Taqman assay employing the primers and probes listed in Table 1. Multiplexed reactions were run for each pair of codons in a single tube in a Rotor Gene 3000 (Qiagen Inc., USA) with four detection channels. The first tube contained primers and probes specific for codons 86 and 1246 and the second tube contained primers and probes specific for codons 184 and 1042. The 25 μl reaction contained 12.5 μl of Rotor-Gene Multiplex PCR Kit mixture (Qiagen Inc., USA), final concentrations of 0.3 μM of each primer, 0.1 μM of each probe and 5 μl of DNA. For detection of codons 86 and 1246, samples were subjected to an initial heating step of 95°C for 5 min followed by 40 cycles of 95°C for 15 s and 52°C for 30 s. For detection of codons 184 and 1042, reactions were subjected to an initial heating step of 95°C for 5 min followed by 40 cycles of 95°C 15 s and 50°C for 30 s. At least two DNA positive controls representing the described codon polymorphisms of *P. falciparum* clones 3D7, Dd2, HB3 and 7G8 were included in each experiment. Reactions with NTC and FP extractions (described above) and with deionized water substituted for template DNA were employed as negative controls in each experiment. Direct sequencing of the Taqman assay products from 45 samples was also performed to verify the polymorphisms in *pfmdr1*.

**Table 1:** **Primers and probes sequences used to amplify and detect SNPs in** ***pfmdr1***

**Position**	**Primer/Probe**	**Sequence 5′→ 3′**
86	Pfmdr1-86F	CCT TTT TTT ATA TCT GTG TTT GGT G
	Pfmdr1-86R	TTA TTA TCA TGA AAT TGT CCA TCT TG
	Pfmdr1-86N	ROX-AAA GAA CAT GAA TTT AGG TGA TGA-BHQ-2a
	Pfmdr1-86Y	Cy5- AAA GAA CAT G**T**A TTT AGG TGA TGA-BHQ-2a
184	Pfmdr1-184F	TTA TAA CAA TTT TTA CAT ATG CCA GTT CC
	Pfmdr1-184R	TCT TAT TAC ATA TGA CAC CAC AAA C
	Pfmdr1-184Y	JOE-TTA GGT TTA TAT ATT TGG TCA TTAAT-BHQ-1a
	Pfmdr1-184F	FAM -TTA GGT TTA T**T**T ATT TGG TCA TTAAT-BHQ-1a
1042	Pfmdr1-1042F	GAA GAA TTA TTG TAA ATG CAG C
	Pfmdr1-1042R	CCT TTT AAG GAC ATT AAT TTT C
	Pfmdr1-1042N	ROX-ATT ATT TAT TAA TAG TTT TGC-BHQ2a
	Pfmdr1-1042D	Cy5-ATT ATT TAT T**G**A TAG TTT TGC-BHQ2a
1246	Pfmdr1-1246F	TGC AGA AGA TTAT ACT GTA TTT AAT A
	Pfmdr1-1246R	GCA AAC TTA CTA ACA CGT TTA ACA
	Pfmdr1-1246D	JOE- TAT AAC TTA AGA GAT CTT AGA AAC-BHQ-1a
	Pfmdr1-1246Y	FAM-TAT AAC TTA AGA **T**AT CTT AGA AAC- BHQ-1a

Mutant bases of the Pfmdr1-86Y, Pfmdr1-184F, Pfmdr1-1042D and Pfmdr1-1246Y primers are shown in bold font. ROX, Cy5, JOE, FAM indicate fluorescent reporter dyes; BHQ-1a and BHQ-2a denote Black Hole Quencher Dyes 1a and 2a, respectively.

A 350 bp region of the *pfnhe1* gene was amplified in 94 of the 99 QN treated samples using nested PCR, as previously described [41]. PCR products were purified by EXOSAP-it (USB, Ohio, USA) and bidirectionally sequenced.

Diversity assessment of 100 randomly selected samples was performed with six polymorphic microsatellite markers present in distinct chromosomes; polyα, TA81, TA1, TA60 and TAA87 [54,55], and 299812 [56]. The reverse primer for each marker was fluorescently labeled and PCR products were analysed in an ABI 3730XL Genetic Analyzer (Life Technologies, Carlsbad, CA, USA). Microsatellite data were analysed with Gene Mapper software (v3.2). A mixed microsatellite haplotype was assigned when the minor signal was at least 33.3% of the major signal [57]. Polyclonality was scored if mixed signals were observed from at least two microsatellites.

## Results

### Parasitaemia and treatment outcomes of children receiving AQ or QN

**Figure 2:**
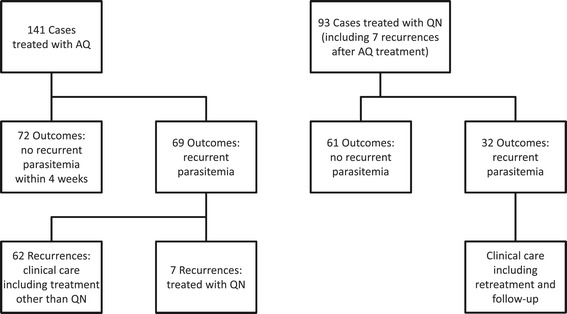
**Flowchart of malaria cases, treatments and outcomes in the Muheza Mother-Offspring Malaria Study.**

Samples available from the Muheza Mother-Offspring Malaria Study included filter paper blood spots from 141 children who presented to the clinic with a new episode of *P. falciparum* parasitaemia, had no history of anti-malarial treatment within the preceding 4 weeks, and were prescribed 3 doses of AQ (Figure 2, see Additional file 1). Of these 141 cases that were treated with AQ, 69 developed microscopic parasitaemia again within four weeks, requiring additional clinical care, while no recurrent parasitaemia was observed in 72 cases. Ninety-three samples were available from cases treated with QN. These samples were from seven cases requiring QN within four weeks of AQ treatment and an additional 86 cases of children presenting with parasite densities ≥ 2,500 pa/200 WBC (est. 100,000 pa/μl; 70 cases) or who were unable to take oral AQ therapy (16 cases; Figure 2).

Presenting parasite densities in the samples from all 141 + 93 = 234 AQ- or QN-treated cases ranged from 40 to 1,119,280 pa/μl with a geometric mean of 30,107 pa/μl (average LN (pa/μl) = 10.313). Geometric means of presenting parasite densities were 11,196 pa/μl (average LN (pa/μl) = 9.323) in the cases treated with AQ and were 134,903 (average LN (pa/μl) = 11.812) in the cases treated with QN (Table 2). Presenting parasite densities ranged from 40 – 96,800 pa/μl in cases that showed no recurrence after AQ treatment and from 4,480 – 1,119,280 pa/μl in cases with no recurrence after QN treatment, while parasite densities in cases of recurrent parasitaemia were 120–204,480 pa/μl after AQ treatment and 1,680 – 744,800 pa/μl after QN treatment. By two-tailed t-tests of LN (pa/μl) average and standard deviation values, there were no significant differences in the geometric mean presenting parasitaemia of cases that did or did not develop recurrent parasitaemia after treatment with AQ (*p* = 0.78) or QN (*p* = 0.37).

**Table 2:** **Clinical data of the AQ-treated and QN-treated groups**

	**AQ-treated group**	**QN-treated group**
Number of episodes	141	93
Mean Hb (g/dl)	10.5	10.6
(range)	(3.0 – 18.8)	(4.5 – 18.3)
Mean Temperature °C	37.6	38.0
(range)	(36.0 – 39.8)	(36.0 – 40.6)
Geometric Mean Parasite density (pa/μl)	11,196	134,903
(range)	(40 – 204,480)	(1,680 - 1,119,280)
Hb S AA	132	86
Hb S AS	7	6
Hb type not determined	2	1

### *pfcrt* haplotypes and treatment outcomes in the Muheza cases

Haplotypes of *pfcrt* were determined for all 234 cases: 219/234 (93.6%) of the samples carried *pfcrt*-CVIET alone, 10/234 (4.3%) carried *pfcrt*-CVMNK alone, and 5/234 (2.1%) carried both of these haplotypes (Table 3). The South American *pfcrt*-SVMNT haplotype was not detected by the Taqman assay or sequencing in any of the samples in this study. By year of collection, the occurrence of *pfcrt*-CVMNK or mixed *pfcrt*-CVMNK/*pfcrt*-CVIET in the samples was 1/32 in 2003 (3.1%), 6/117 in 2004 (5.1%), 8/76 in 2005 (10.5%) and 0/9 in 2006 (0.0%). The different percentages of *pfcrt*-CVMNK haplotypes in these samples were not statistically significant for a trend between 2003 and 2006 (chi-square test for trend *p* = 0.27).

**Table 3:** **Distribution of PfCRT and PfMDR1 haplotypes in the study population 2003 – 2006**

	**Year**				
	**2003**	**2004**	**2005**	**2006**	**TOTAL**
**PfCRT amino acid positions 72–76**					
CVMNK only	0.0% (0)	3.4% (4)	7.9% (6)	0% (0)	**4.3% (10)**
CVIET only	96.9% (31)	94.9% (111)	89.5% (68)	100% (9)	**93.6% (219)**
CVMNK & CVIET mixed	3.1% (1)	1.7% (2)	2.6% (2)	0% (0)	**2.1% (5)**
*Number of samples*	*32*	*117*	*76*	*9*	***234***
**PfMDR1 amino acid positions 86, 184, 1042, 1246**					
YYNY only	67.7% (12)	67.8% (59)	66.2% (43)	75% (6)	**67.4% (120)**
YYNY mixed*	5.6% (1)	11.5% (10)	9.2% (6)	0.0% (0)	**9.6% (17)**
YYND only	16.7% (3)	10.3% (9)	13.8% (9)	12.5% (1)	**12.4% (22)**
YYND mixed**	0.0% (0)	10.3% (9)	7.7% (5)	0.0% (0)	**7.9% (14)**
Other haplotypes	11.1% (2)	6.9% (6)	9.2% (6)	12.5% (1)	**8.4% (15)**
*Number of samples*	*18*	87***	65***	*8*	178***

*Mixed with codons of other haplotypes, including 10 YYND (6 in 2004; 4 in 2005).

**Mixed with codons of other haplotypes, including 10 YYNY (6 in 2004; 4 in 2005).

***Total is corrected for the YYNY + YYND mixed samples that are entered twice, as both YYNY mixed and as YYND mixed samples.

Among the cases prescribed AQ and carrying the *pfcrt*-CVIET or mixed *pfcrt*-CVMNK/*pfcrt*-CVIET haplotype, only 70/137 (51.1%) remained clear of recurrent parasitaemia for more than four weeks. Similarly, only 2/4 (50%) of cases receiving AQ and carrying *pfcrt*-CVMNK alone remained clear of recurrent parasitaemia for more than four weeks. QN treatment of infections carrying the *pfcrt*-CVIET or mixed *pfcrt*-CVMNK/*pfcrt*-CVIET haplotype cleared 57/87 (65.5%) of the parasitaemia for more than four weeks. Similarly, 4/6 (66.7%) of infections receiving QN and carrying the *pfcrt*-CVMNK haplotype alone remained clear of recurrent parasitaemia for more than four weeks.

### Distribution of *pfmdr1* haplotypes

From the 234 samples described above, 178 samples were successfully assessed for four *pfmdr1* codon polymorphisms: N86 or 86Y; Y184 or 184F; N1042 or 1042D; D1246 or 1246Y (Table 3, Additional file 1). Codons of the *pfmdr1-*YYNY haplotype either alone or mixed with polymorphisms of other *pfmdr1* alleles occurred in 137/178 (77.0%) of the samples. Further analysis showed that the codons of *pfmdr1-*YYNY were present in 135/169 (79.9%) of the *pfcrt-*CVIET-containing samples but in only 2/9 (22.2%) of samples containing *pfcrt-*CVMNK alone: this difference was found to be statistically significant (Fishers’ exact *p* < 0.001). In contrast, there was no evident association between *pfmdr1*-YYND and *pfcrt*-CVIET (Fisher’s Exact *p* = 0.63). The *pfmdr1-*86Y polymorphism occurred either alone or mixed with *pfmdr1*-N86 in 163/178 (91.6%) samples, while *pfmdr1-*1246Y occurred either alone or mixed with *pfmdr1*-D1246 in 146/178 (82.0%) samples. The wild-type *pfmdr1-*Y184 codon was detected in 174/178 (97.8%) samples. All 178 isolates carried the wild-type *pfmdr1-*N1042 polymorphism.

Of the 178 samples typed for *pfmdr1* polymorphisms, 95 were from children who received AQ treatment. AQ cleared 47/95 (49.5%) of these infections with no recurrence of parasitaemia for more than four weeks. The *pfmdr1-*86Y polymorphism was detected in 45/47 (95.7%) of children who remained clear of parasitaemia and in 45/48 (93.8%) of the children who developed recurrent parasitaemia within four weeks (Fishers’ exact *p* = 1.00). The *pfmdr1*-1246Y polymorphism was detected in 38/47 (78.7%) of children who remained clear of parasitaemia and in 40/48 (83.3%) of the children who developed recurrent parasitaemia within four weeks; this difference was also not significant (Fishers’ exact *p* = 0.79).

Eighty-three of the 178 samples typed for *pfmdr1* polymorphisms were from children who received QN treatment. QN cleared 56/83 (67.5%) of these infections with no recurrence of parasitaemia for more than four weeks. The *pfmdr1*-86Y polymorphism was present in 49/56 (87.5%) of children who remained clear of parasitaemia and in 24/27 (88.9%) of the children who developed recurrent parasitaemia within four weeks (Fishers’ exact *p* = 1.00). The *pfmdr1*-1246Y polymorphism was present in 46/56 (82.1%) infections of children who remained clear of parasitaemia and in 22/27 (81.5%) of the children who developed recurrent parasitaemia within four weeks; this difference was not significant (Fishers’ exact *p* = 1.00).

### *pfnhe1* ms-4760 repeat haplotypes in cases treated with QN

**Figure 3:**
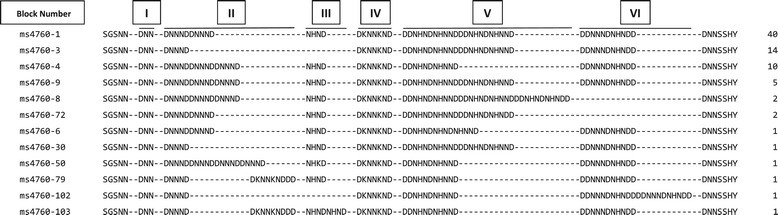
**Predicted amino acid sequences from different** ***pfnhe1*** **ms-4760 haplotypes in Muheza.** The alignment includes repeats of the *pfnhe1* ms-4760 microsatellite in 79 samples. Left column: ms-4760 designation; center column: translated sequences from *pfnhe1* microsatellite repeat region including blocks I–VI [45]; right column: number of samples in which each haplotype was observed.

Of the 93 cases treated with QN, a 350 bp region of the *pfnhe1* gene containing the ms-4760 repeat region was successfully amplified and sequenced from 79/93 (85%) sample preparations. Twelve different haplotypes of ms-4760 were observed, including two previously unreported haplotypes (ms-4760-102 and ms-4760-103; Figure 3). The most prevalent haplotype was ms-4760-1 (two DNNND repeats in block II), observed in 40/79 (50.6%) of the samples; the second most frequent haplotype, ms-4760-3 (one DNNND repeat in block II) occurred in 17.7% (14/79) of samples. Sequences of the observed haplotypes and the number of samples in which each was detected are presented in Figure 3.

Of the 79 QN-treated cases typed for ms-4760, 30.4% (24/79) had returned with parasitaemia within four weeks of treatment, while 69.6% (55/79) remained clear of parasitaemia. The presence of two or more repeats of DNNND in ms-4670 was not a predictive factor for the return of parasitaemia within 28 days (Fishers’ exact *p* = 0.77). Likewise, there was no association of two or more *vs* only one DDNHNDNHNND repeat with return of parasitaemia after the QN treatment (Fishers’ exact *p* = 1.0).

### Population diversity

Four polymorphic microsatellites that reside near anti-malarial drug resistance loci (MS-Polyα, chromosome 4; MS-TAA81, chromosome 5; MS-299812, chromosome 8; MS-TA60, chromosome 13), and 2 microsatellites of chromosome 6 (MS-TA1 and MS-TAA87) were typed in 100 randomly selected samples. For a given microsatellite, a mixed haplotype was assigned if the fluorescence peak from the minor band was at least 33% of the signal from the major band.

PCR assays of the Polyα, MS-TAA81, MS-299812 and MS-TA60 microsatellites detected 28, 19, 19 and 13 size polymorphisms, respectively. For MS-TA60 on chromosome 13, a 70 bp band was obtained from 41.6% of infections in either a single or mixed haplotype. On chromosome 6, assays of MS-TAA87 detected 38 major and 23 minor size polymorphisms, whereas assays of MS-TA1 detected 23 major and 13 minor polymorphisms. Most infections in the population differed from each other at two or more microsatellites, consistent with a high level of polyclonality and high transmission rates in this area of Tanzania.

## Discussion

Related mechanisms of drug action and resistance are reflected in the structural similarities of CQ and AQ as well as the evidence that *pfcrt* polymorphisms associated with CQ resistance affect AQ efficacy. In a study of *pfcrt* haplotypes and MDAQ responses *in vitro,* Warhurst *et al.* [58] proposed that AQ resistance is associated with the hydrophobicity of *pfcrt*-SVMNT polymorphisms. Amino acid changes encoded by *pfmdr1* that modulate responses of CQ-resistant parasites have also been shown to affect responses to AQ and MDAQ [24]. In the analysis of *pfcrt* and *pfmdr1* of two *P. falciparum* crosses, Sa *et al.* [5] found that the highest *in vitro* IC_50_ levels for MDAQ occurred in *pfcrt*-CVIET or *pfcrt*-SVMNT progeny carrying either *pfmdr1*-NFCDY or *pfmdr1*-NFSDD (codon positions 86, 184, 1034, 1042, 1246), consistent with the association of the *pfcrt*-SVMNT/*pfmdr1*-NFCDY combination with AQ failure in South America. These findings together with reports of the *pfcrt*-SVMNT haplotype in Tanzania, Angola, and Cameroon, raise the concern that if *pfmdr1* with the 1042D change spread into Africa it could promote high-level AQ resistance where this drug is widely deployed as a partner drug in ACT combinations [50].

In the present study, 234 samples collected over a four-year period from Muheza, Tanzania were analysed for the presence of *pfcrt* polymorphisms. The mutant *pfcrt*-CVIET (Southeast Asian CQ-resistant form) predominated in the parasite population while the wild type (CQ-sensitive) *pfcrt*-CVMNK form was present in 1/32 (3.1%) of samples in 2003, 6/117 (5.1%) in 2004, 8/76 (10.5%) in 2005 and 0/9 (0%) in 2006; there was no statistically significant trend for *pfcrt*-CVMNK prevalence among these samples over the years. The *pfcrt*-SVMNT haplotype was not detected in any of the samples analysed in this study. A high level of genetic diversity among all samples was confirmed by six highly polymorphic microsatellites of chromosomes 4, 5, 6 (two markers), 8 and 13. This diversity is an expected outcome in high transmission areas such as Muheza where the entomological inoculation rate has been reported to be 405 infectious bites per year [59].

The *pfmdr1*-1042D polymorphism that is able to greatly affect MDAQ susceptibility has not been reported in Africa so far; however, three studies on the occurrence of *pfcrt*-SVMNT in Africa did not provide data for *pfmdr1* haplotypes [47-49]. The present study included assessments of 178 Muheza samples for *pfmdr1* polymorphisms at codon positions 86, 184, 1042 and 1246. All 178 samples were found to encode only *pfmdr1-*N1042 with no evidence for the presence of *pfmdr1-*1042D. Consistent with previous suggestions that *pfcrt* and *pfmdr1* polymorphisms in Asia and Africa may be maintained by substantially different mechanisms than in South America [60], the *pfmdr1*-1042D amino acid change may be slow to rise in African *P. falciparum* populations because of genetic background or other factors that are less supportive for this haplotype than in South America. The impediment posed by these factors remains open to question, however, as laboratory experiments have shown that the N1042D codon change can be crossed into the African background (as demonstrated by results from the 7G8/Brazil × GB4/Ghana cross [5]). High prevalence in this study of the major *pfmdr1*-YYNY haplotype in *pfcrt*-CVIET-carrying parasites is consistent with previous findings of linkage disequilibrium between *pfcrt*-76T and *pfmdr1*-86Y polymorphisms [61-63].

The diversity of the *pfnhe*1-ms-4760 repeat in Muheza is similar to the diversity reported from other regions where this repeat has been studied [41,45,46]. The ms-4760-2 haplotype was not detected, consistent with the absence of this haplotype from a previous report from East Africa [46]. However, ms-4760-4 was observed in ten samples, ms-4760-72 in two samples and ms-4760-79 in one sample; these forms of ms-4760 were not found in previous work from East Africa [46] or in a recent study from Mali [41]. In addition, two haplotypes (ms-4760-102 and ms-4760-103), which have not been previously reported, were observed. A recent report found an association of three DNNND repeats in ms-4760 and higher IC_50_ for QN in Western Kenya [64]. The results of the present study did not show this association of three DNNND repeats in cases of returned parasitaemia after QN treatment.

Several possible explanations may account for the frequent recurrence of a parasitaemia within 28 days of treatment and the lack of clear associations to known *pfcrt*, *pfmdr1* and *pfnhe1* markers of AQ and QN resistance. These include the high entomological inoculation rates that may have contributed frequent reinfections from emergent liver forms as drug levels wane after treatment. The extent of clearance of moderately-resistant parasites carrying *pfcrt*-CVIET*, pfmdr1-*86Y, *pfmdr1*-1246Y or *pfnhe1* haplotypes of ≥ 2 DNNND repeats may have been affected by the immune status of the children [65]. Finally, the authors note that anti-malarial treatments were not fully supervised and plasma drug levels were not followed as part of the parent study to this sub-study; unless disease severity warranted in-patient treatment, children were released from the clinic with medicine and instructions to the parents to complete the prescribed treatment. Compliance and pharmacokinetics of the drugs may have affected treatment efficacy, as has been reported elsewhere [66-69].

Previous studies have documented the return of the wild-type *pfcrt*-CVMNK haplotype of CQ-sensitivity in parasite populations where alternative drugs including ACT replaced CQ [7-18]. Tanzania changed its malaria treatment policy from CQ to SP in 2001. Although a statistically significant increase of *pfcrt*-CVMNK in Muheza over the period was not found in this study, the presence of this wild-type form in the region may set the stage for the return of increased prevalence of the *pfcrt* wild-type, as CQ-sensitive parasites in the absence of selective pressure have been observed elsewhere in Tanzania where CQ therapy has also been removed [11]. Selection of opposite alleles of *pfmdr*1 by ACT partner drugs, such as AQ and lumefantrine [70], suggest that these drugs could be alternated in order to prolong their efficacy.

Occurrence of the *pfcrt*-S_agt_VMNT haplotype in Africa has been reported at low prevalence in countries other than Tanzania: examples include instances of this haplotype in Ghanaian samples collected in 1996-97 [60] and recently from Cameroon [48]. Disparate findings can be found in reports from Angola in 2007 and 2010, where *pfcrt*-S_tct_VMNT was detected in 58% of 102 samples from Luanda [49], but there was a complete absence of *pfcrt*-S_tct_VMNT from 430 samples collected in Dande [71]. Considering that Luanda and Dande are approximately 90 km apart, additional surveys will be needed to establish the potential prevalence of *pfcrt*-S_tct_VMNT in this region.

## Conclusions

Although the *pfcrt*-SVMNT haplotype associated with amodiaquine resistance has been reported from Korogwe, Tanzania, this study of 234 samples from Muheza, approximately 40 kilometers away and in the same time period, did not detect this haplotype. The *pfcrt*-CVIET haplotype widely responsible for chloroquine resistance was most prevalent in this study and was significantly associated with the *pfmdr1*-YYNY haplotype. Low levels of the wild-type *pfcrt*-CVMNK haplotype in Muheza may set the stage for the return of chloroquine sensitive *P. falciparum* populations, as has been reported in other regions where chloroquine use was stopped. Relative absence of *pfcrt*-SVMNT from Africa may reflect genetic or other factors affecting *P. falciparum* that are less supportive for this haplotype than in South America and other continental regions.

## Additional file

Additional file 1:
**Clinical information, treatment outcomes and haplotypes of** ***pfcrt*** **,** ***pfmdr1 and pfnhe1*** **from children in the Muheza Mother-Offspring Malaria Study.**
